# External validation of an early warning alert for elevated intracranial pressure in the Avert-IT database

**DOI:** 10.1186/cc13643

**Published:** 2014-03-17

**Authors:** M Beckers, F Güiza, B Depreitere', I Piper, R Donald, G Van den Berghe, G Meyfroidt

**Affiliations:** 1 University Hospitals Leuven, Belgium; 2 Southern General Hospital, Glasgow, UK; 3 University of Glasgow, UK

## Introduction

After severe traumatic brain injury (TBI), episodes of elevated intracranial pressure (ICP) are associated with poor outcome. Previously we developed a model to predict increased ICP, 30 minutes in advance, using the dynamic characteristics of routinely monitored minute-by-minute ICP and mean arterial blood pressure (MAP) signals [1]. The model was developed using data from the Brain-IT database [2]. Here we present external validation results of this model, on a more recent cohort of adult TBI patients from the AVERT-IT project [3].

## Methods

A retrospective analysis of physiological data collected at the minute resolution, from 43 adult patients from the AVERT-IT project. A total of 67 episodes of ICP above 30 mmHg lasting at least 10 minutes were identified in this cohort. Four-hour time series of ICP and MAP anteceding each episode by 30 minutes were analyzed. Additional time series not preceding elevated ICP episodes were used for validation.

## Results

Table 1 summarizes the main findings. Performance of the model in the original study [1] is reproduced in the first column. The model retains identical performance for all criteria in the cohort of more recent TBI patients of the AVERT-IT database.

**Table 1:** 

Database	Brain-IT	Avert-IT
AUROC	0.85	0.83
aBrier SS (%)	34	30
Cal large	0.00	0.01
Cal slope	0.99	1.03
Accuracy (%)	77	77

## Conclusion

The obtained external validation results, on a previously unseen cohort of adult TBI patients, confirm the robustness of the model to accurately predict future increased ICP events 30 minutes in advance. The general applicability of the model is probably due to its sparseness, as it only uses two routinely monitored signals as input, namely ICP and MAP. These results are a large step forward in our work toward an early warning system for elevated ICP that can be used worldwide.
